# Comparative Genomics Analysis Demonstrated a Link Between Staphylococci Isolated From Different Sources: A Possible Public Health Risk

**DOI:** 10.3389/fmicb.2021.576696

**Published:** 2021-02-25

**Authors:** Rory Cave, Raju Misra, Jiazhen Chen, Shiyong Wang, Hermine V. Mkrtchyan

**Affiliations:** ^1^School of Health, Sport and Bioscience, University of East London, London, United Kingdom; ^2^Natural History Museum, Core Research Laboratories, Molecular Biology, London, United Kingdom; ^3^Department of Infectious Disease, Huashan Hospital, Fudan University, Shanghai, China; ^4^School of Biomedical Sciences, University of West London, London, United Kingdom

**Keywords:** coagulase-negative staphylococci, comparative genomics, public settings, phylogenetics, public health

## Abstract

Coagulase-negative staphylococci (CoNS) have been recovered from different ecological niches, however, little is known about the genetic relatedness of these isolates. In this study, we used whole genome sequencing to compare *mecA* positive (*mecA*^+^) *Staphylococcus epidermidis*, *Staphylococcus haemolyticus* and *Staphylococcus hominis* isolates recovered from hand-touched surfaces from general public settings in East and West London with data of isolates deposited to European Nucleotide Archive (ENA) by other research groups. These included isolates associated with hospital settings (including those recovered from patients), healthy humans, livestock, pets, plants and natural, and other public environments. Using core and accessory phylogenetic analyses we were able to identify that the *mecA^+^ S. epidermidis* and *S. haemolyticus* isolates recovered from general public settings were genetically related to isolates recovered from the bloodstream, urinary tract and eye infections. *S. epidermidis* isolates recovered in our study were also shown to be genetically related to isolates previously recovered from livestock/livestock housing, whereas *S. haemolyticus* isolates were genetically related to isolates recovered from a dog and kefir (fermented cow milk drink). *MecA*^+^ *S. hominis* isolates were not genetically related to any isolates recovered from clinical samples but were genetically related to isolates recovered from mosquitoes, air samples (residential areas) and kefir. All three species showed to have genetic relatedness to isolates recovered from healthy humans. These results show that CoNS isolates in this study share genetic similarities with those of different lineages and that *mecA^+^ S. epidermidis* and *S. haemolyticus* isolates found in general public settings in this study may pose a risk to public health.

## Introduction

Hand touched surfaces in public areas act as an intermediate for human to human transmission of pathogenic bacteria (Lei et al., 2017). Many bacteria responsible for various infections are known to have originated from human and animal sources which later transmitted across the species barrier (Fitzgerald, 2012; Argudín et al., 2015b). However, little is known of the genetic lineages of coagulase-negative staphylococci (CoNS) recovered from hand touched surfaces in public settings and the threat they pose to public health.

Coagulase-negative staphylococci are the most common commensal group of bacteria found on human skin and frequently found on surfaces in hospitals, hotel rooms, libraries, university campus and public transport (Becker et al., 2014; Seng et al., 2017; Xu et al., 2018b; Cave et al., 2019). Unlike coagulase-positive *Staphylococcus aureus* they lack many key virulent factors; however, *Staphylococcus epidermidis*, *Staphylococcus haemolyticus* and *Staphylococcus hominis* have been identified as significant pathogens associated with nosocomial infections and medical devices (Cherifi et al., 2013; Chaves et al., 2005; Czekaj et al., 2015). *S. epidermidis* accounts for 22% of bloodstream infections in intensive care unit patients in the United States, whereas CoNS accounts for 23.1 and 12.7% of bloodstream infections in Israel and China, respectively (Otto, 2009; Abu-Saleh et al., 2018; Cui et al., 2019). In addition, CoNS have also been reported to be associated with community and animal/livestock infections (Nanoukon et al., 2017; Schoenfelder et al., 2017). Many CoNS infections are challenging to treat due to them being resistant to multiple antibiotics (Lee et al., 2018; Cui et al., 2019; Pain et al., 2019). One antibiotic resistance gene in particular which makes infections caused by staphylococci challenging to treat is *mecA*. This gene confers resistance to all beta-lactam antibiotics, including carbapenem, cephalosporin, penam, cephamycin and monobactam and is associated with the “superbug” methicillin-resistant *S. aureus* (MRSA), a bacterium that has caused severe infections in healthcare settings, community and livestock worldwide.

London is the most densely populated city in Europe, with a population of 8.7 million people (2016) and a population density of 5,590 per km^2^ (2016) (Land Area and Population Density, Ward and Borough - London Datastore, 2020). In 2018, it was estimated that there were 60,788 cases of antibiotic-resistant severe infections in England with London having the highest rate of bloodstream infections caused by antibiotic resistant bacteria (42.9 per 100,000 population) (English Surveillance Programme for Antimicrobial Utilisation and Resistance (ESPAUR), 2019). In addition, CoNS can be commonly isolated from environmental sites in London; with one study showing that 94% of the recovered CoNS isolates were antibiotic-resistant and 11% carrying the *mecA* gene (Xu et al., 2018b).Currently, due to the improvement of whole genome sequencing (WGS) technology, it is possible to determine the genetic relationship of less frequently studied bacteria from different settings (Quainoo et al., 2017).

In this study, we genetically compared *mecA* positive (*mecA*^+^) *S. epidermidis*, S. *haemolyticus* and *S. hominis* isolates recovered from high-frequency touched surfaces of general public settings in the community and public areas in hospitals with complete and draft genomes [obtained from the European Nucleotide Archive (ENA)] of the isolates recovered from different sources, including bloodstream, urinary tract, and eye infections, healthy humans, livestock, pets, plants, fermented milk drink, natural and other public environmental sites. This comparative genomics analysis helped to assess whether *mecA*^+^ CoNS isolates recovered in our study were genetically similar to isolates recovered from different sources (obtained from the ENA), including those that have previously been reported to cause infections.

## Materials and Methods

### Sample Collection

Staphylococcal isolates were recovered between November 2016 to September 2017 from high-frequency hand touched surfaces of inanimate objects from two locations in East London and two locations in West London. These locations included one area considered as public settings (shopping centres and train stations) and another area within hospitals where the general public had easy access, without being a patient (reception area, public washrooms, corridors and lifts). Fifty sites from each location were randomly sampled using COPAN dry swabs (Copan Diagnostics Inc., United States). Six hundred staphylococcal isolates were recovered of which 224 were from East London and 376 from West London. One hundred and eighty-two of the isolates were from the community area and 418 from hospital areas. Ninety-seven of the isolates were from East London community area; 85 from West London Community area; 127 from East London Hospital (ELH) and 376 from West London Hospital (WLH) (Cave et al., 2019).

### Isolation of Staphylococci

All samples were directly inoculated onto mannitol salt agar (MSA, Oxoid Basingstoke, United Kingdom) within 1–3 h of recovery and incubated aerobically for 24–72 h at 37°C. To prevent bias up to 10 colonies from each plate were picked each having different colony morphology or if there are less than 10 different colony morphologies an equal amount of different colony morphologies was selected. These isolates were screened for potential staphylococci characteristics, including performing catalase and coagulase tests. Prolex^TM^ staph latex kit (ProLab Diagnostics, Neston, United Kingdom) was used to distinguish *S. aureus* and CoNS (Cave et al., 2019).

### Identification of Staphylococci Recovered From High-Frequency Hand Touch Areas

Staphylococcal isolates were initially identified by gram staining and catalase test. The presumptive staphylococcal isolates were further identified at species level using Matrix-assisted laser desorption ionization-time of flight mass spectroscopy (MALDI-TOF-MS, Microflex LT, Bruker Daltonics, Coventry, United Kingdom) in a positive linear mode (2000–20,000 m/z range). Samples were prepared using a full protein extraction method as described previously (Cave et al., 2019).

### Detection of the *mecA* Gene by PCR

Forty three bacterial isolates DNA was extracted via boil lysis and used to perform PCR for detection of the *mecA* gene as previously described (Cave et al., 2019). Using the Met1 and Met2 primers (Eurofins, Germany) PCR reactions were performed in a 20 μl volume (Hanssen et al., 2004), consisting of 10 μl of Phusion Master Mix;1 μl of forward primer, 1 μl of reverse primer, 7 μl of sterile distilled water and 1 μl of isolates DNA template. The PCR condition for this reaction was 94°C for 5 min followed by 35 cycles of denaturation at 94°C for 30 s, annealing at 52°C for 30 s and extension at 72°C for 1 min with a final extension at 72°C for 10 min.

### Genome Sequencing and Assembly

Whole genome sequencing was performed for 43 *mecA*^+^ staphylococci isolates using Illumina HiSeq platform. Seven out of 43 isolates were whole genome sequenced by MicrobesNG (Birmingham, United Kingdom) and the remaining isolates were sequenced at Fudan University, Shanghai, China (Supplementary Table 1).

Genomic DNA was extracted using TIANamp Bacteria DNA kit (Tiangen, China) and paired-end sequencing libraries were constructed using Nextera XT DNA Sample Preparation kits or TruSeq DNA HT Sample Prep Kit (Illumina, United States) following manufacturer’s instruction.

Quality of reads was assessed using FASTQC and trimmed using trimmomatic (Version 0.35), default settings, specifying a Phred cut-off of Q20 (Andrews, 2011; Bolger et al., 2014). Trimmed reads were *de novo* assembly by SPAdes 3.11 and contig assembly was analyzed by QUAST and contigs that were ≤500 bp were removed (Bankevich et al., 2012; Gurevich et al., 2013). The species of these isolates were confirmed by 16S rRNA sequencing from the assembled genomes by using the barrnap software^1^ and searched against a database of known 16S rRNA sequences using NCBI BLAST tool with a cut-off for species identity of 95% similarity (Altschul et al., 1990; Janda and Abbott, 2007).

### Phylogenetic Analyses

A core single nucleotide polymorphism (SNP) maximal likelihood tree was constructed using isolates previously recovered from different sources (data obtained from the ENA, accessed in July 2019; Supplementary Table 2). The main selection criteria of the isolates obtained from ENA database was their isolation sources, which have been recorded in the ENA and/or reported in a peer-reviewed literature. The selection criteria for isolates recovered in this study was the presence of clinically important *mecA* gene. SMALT version 0.5.8^2^ was used to map short reads against reference genomes. Reference genomes used to map each staphylococcal species included *S. epidermidis* ATTC 11228; *S. haemolyticus* JCSC 1435 and *S. hominis* K1. SNP calling was done in parallel with all samples of the same species using VarScan version 2.3.9 (Koboldt et al., 2009). VCF file was converted to multi-FASTA alignment file using the python script vcf2phylip^3^. Recombination was detected and removed from the genome with Gubbins (Croucher et al., 2015). A maximal likelihood tree was constructed using RAxML version 8 using the generalised time reversible model (GTR) model with GAMMA method of correction for site rate variation and 100 bootstrap replications (Stamatakis, 2014). The phylogenetic tree was visualised and annotated using ITOL (Letunic and Bork, 2016).

The distance of the accessory genome for each isolate was determined using the variable-length *k*-mer comparisons to distinguish isolates’ divergence in shared sequence and gene content using the POPpunk pipeline (Lees et al., 2019). The number of mixture components was adjusted for each species for obtaining a low-density score (proportion of edges in the network), high transitivity score and high overall score (network score based on density and transitivity). Accessory genome distance was determined by t-SNE with the perplexity (the number of close neighbours each point has) was adjusted for each species to provide the clearest picture of clustering and visualised using Microreact (Argimón et al., 2016).

### Identifying the Unique Accessory Genes That Were Only Present in the ENA *S. hominis* Clinical Isolates

To determine the unique accessory genes in *S. hominis* hospital associated isolates obtained from the ENA, pangenome analysis was performed using Roary pipeline version 3.4.2. We then compared the differences in genes between all *S. hominis* hospital associated isolates obtained from the ENA with the rest of the *S. hominis* isolates used in phylogenetic analyses (Page et al., 2015).

## Results

### CoNS *mecA* From Public Settings

The *mecA* gene was identified in 43 isolates that were recovered from high frequency touched surfaces from general public settings from the community and public areas in hospitals (Table 1). This included: *S. epidermidis* (*n* = 17); *S. haemolyticus* (*n* = 10), *S. hominis* (*n* = 10), S*taphylococcus cohnii* (*n* = 3) and *Staphylococcus warneri* (*n* = 3).

**TABLE 1:** *mecA*^+^ coagulase negative staphylococcal isolates recovered from East and West London public settings.

Isolate ID	Species (*mecA*^+^)	Public settings in London
1	*S. haemolyticus*	ELC
93	*S. haemolyticus*	ELC
99	*S. haemolyticus*	ELC
105	*S. haemolyticus*	ELC
207	*S. hominis*	WLC
208	*S. hominis*	WLC
209	*S. hominis*	WLC
211	*S. cohnii*	WLC
321	*S. epidermidis*	ELH
327	*S. epidermidis*	ELH
329	*S. epidermidis*	ELH
343	*S. cohnii*	ELH
349	*S. cohnii*	ELH
355	*S. epidermidis*	ELH
361	*S. haemolyticus*	ELH
372	*S. hominis*	ELH
373	*S. haemolyticus*	ELH
385	*S. hominis*	ELH
386	*S. hominis*	ELH
387	*S. hominis*	ELH
407	*S. epidermidis*	ELH
435	*S. epidermidis*	WLH
436	*S. epidermidis*	WLH
445	*S. haemolyticus*	WLH
465	*S. epidermidis*	WLH
475	*S. epidermidis*	WLH
479	*S. hominis*	WLH
492	*S. haemolyticus*	WLH
506	*S. haemolyticus*	WLH
538	*S. haemolyticus*	WLH
620	*S. hominis*	WLH
623	*S. hominis*	WLH
631	*S. epidermidis*	WLH
664	*S. epidermidis*	WLH
673	*S. epidermidis*	WLH
699	*S. warneri*	WLH
700	*S. warneri*	WLH
702	*S. warneri*	WLH
711	*S. epidermidis*	WLH
712	*S. epidermidis*	WLH
713	*S. epidermidis*	WLH
715	*S. epidermidis*	WLH
716	*S. epidermidis*	WLH

*ELC, East London Community; WLC, West London Community; ELH, East London Hospital; WLH, West London Hospital.*

### Phylogenetic Analysis of *S. epidermidis*

**FIGURE 1:**
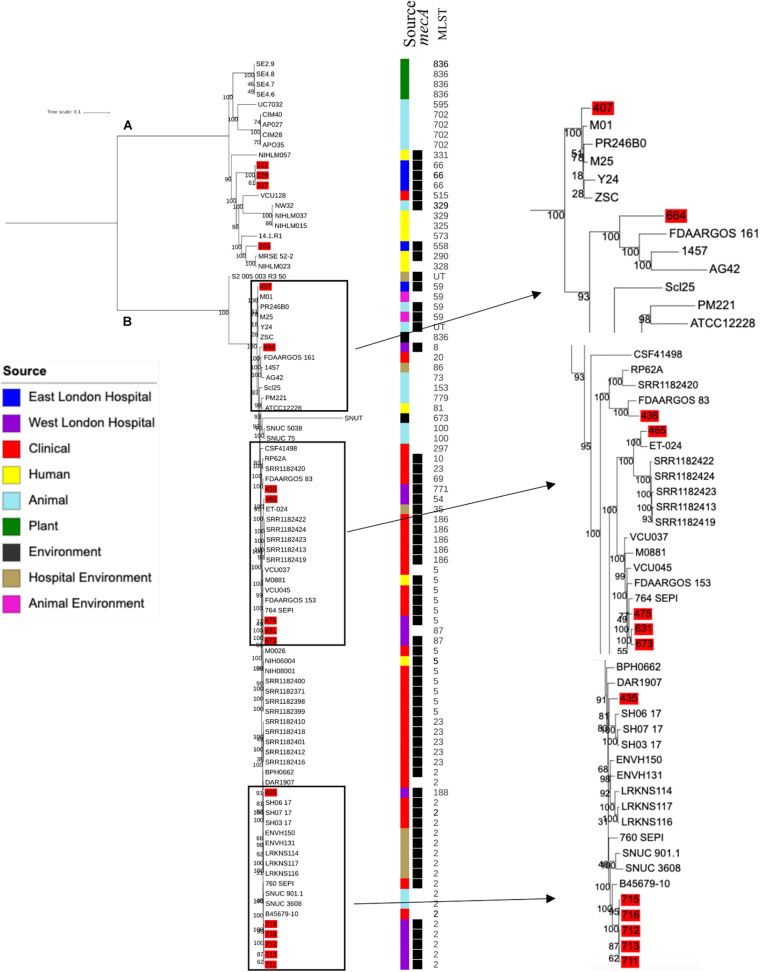
Core SNP maximum likelihood phylogenetic tree of 90 *S. epidermidis*, including isolates recovered from public settings in this study and those obtained from the ENA. **(A,B)** represent the two most distinct clades. Isolates recovered from public settings in London are highlighted in red. MLST = sequence type, UT = untypable.

**FIGURE 2:**
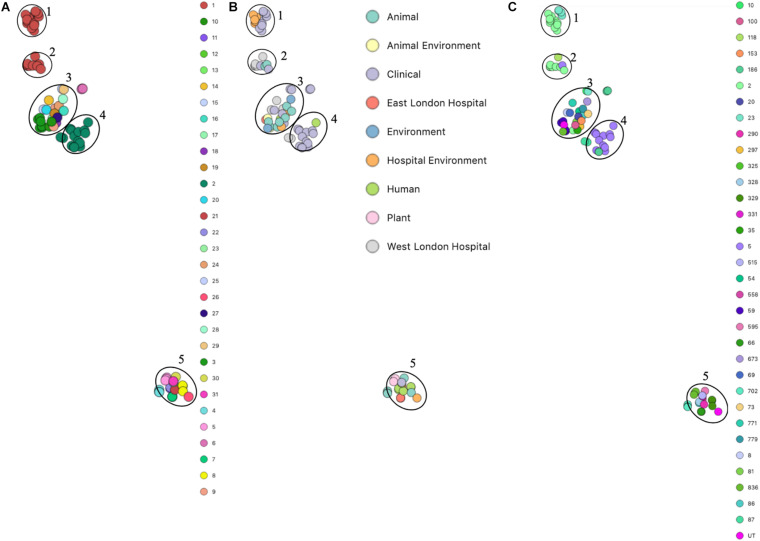
PopPUNK t-SNE analyses of accessory genomes of 90 *S. epidermidis* isolates. Analyses were performed using PopPUNK pipeline. A maximum number of mixture components were set at 5 and for the perplexity of t-SNE set at 20. **(A)** Combined core and accessory genome cluster from PopPUNK analysis; **(B)** Isolation source and **(C)** MLST.

Phylogenetic analysis was performed to determine the relatedness of environmental isolates in this study with those recovered from other sources, including isolates recovered from infections (ENA). In this study, 17 *S. epidermidis mecA^+^* isolates recovered from East and West London were compared to those obtained from the ENA that have previously been recovered from infections (*n* = 34); healthy humans (*n* = 9), livestock (*n* = 13) rodents (*n* = 2), plants (*n* = 4), hospital environment (*n* = 7), animal housing environments (*n* = 2) and natural environments (*n* = 2). Core SNP phylogenetic tree analysis identified two distinctive clades of which 59 out of 90 were *mecA*^+^ (Figure 1). Four isolates from ELH (321, 327, 329 and 355) belonged to clade A, whereas 1 isolate (407) recovered from ELH was in clade B together with all (*n* = 12) isolates recovered from WLH. Interestingly, all ENA isolates recovered from infections, except for VCU128 which was recovered from human airways, were found to be in clade B. ENA isolates recovered from healthy humans and animals were found within both clades whereas those recovered from plants (all rice seeds) were found in clade A only. Isolate 355 recovered form public settings in our study was genetically related to those ENA isolates that have been recovered from healthy humans (MRSE 52-2 and NIHLM057); isolate 407 was genetically related to those isolates that have been recovered from cow (Y24), pig (PR246B0) and animal housing (M01 and M025), whereas isolates 435, 436, 465, 475, 631, 673, 711, 712, 713, 715 and 716 from WLH were genetically related to isolates previously recovered (ENA) from bloodstream (B45679, 764 SEPI, FDARGOS 153, FDARGOS 83, VCU045, SH06 17 and SH03 17, SH7 17) and an endotracheal tube biofilm of a mechanically ventilated patient (ET-0240). Isolates 321, 327 and 329 recovered from ELH uniquely showed no relatedness to any other isolate. PopPUNK analyses revealed that *S. epidermidis* isolates can be combined into 31 groups by their combined core and accessory genome. The accessory genome t-SNE analyses, set at the perplexity of 20, showed that there were five distinct clusters (Figure 2). Two of these groups had a mixture of isolates belonging to different combined clusters. None of the clusters includes isolates belonging to a single sequence type. In this study, the *mecA*^+^ *S. epidermidis* isolates recovered from East and West London were found in different clusters. The accessory genome of *mecA^+^ S. epidermidis* isolates recovered from public settings was related to those ENA isolates that have been previously recovered from bloodstream infections (B45679-10, FDAARGOS 83 and FDAARGOS 161), infected airways (VCU45 and VCU128) and cerebrospinal fluid (CSF41498), endotracheal tube biofilm of a mechanically ventilated patient (ET-024), central venous catheter (1457); from healthy human [skin (914.1.R1), mucosa (ATCC 12228) and human airways (MRSE 52-2)]; from livestock [cows (SNUC 901, SNUC 3608, SNUC 75, SNUC, Y24, PM221 and NW32), a pig (PR246B0) and a sheep (AG42)], a mouse (SCL25); plants (SE2.9, SE4.8, SE4.7 and SE4.6) and from a natural environment (SNUT). In addition, we found that cluster 1 included ENA isolates that were recovered from hospital environments in medical wards and isolates recovered from bloodstream infections, whereas cluster 4 included isolates all recovered from the bloodstream and only a single isolate from healthy human skin (M008).

### Phylogenetic Analyses of *S. haemolyticus*

**FIGURE 3:**
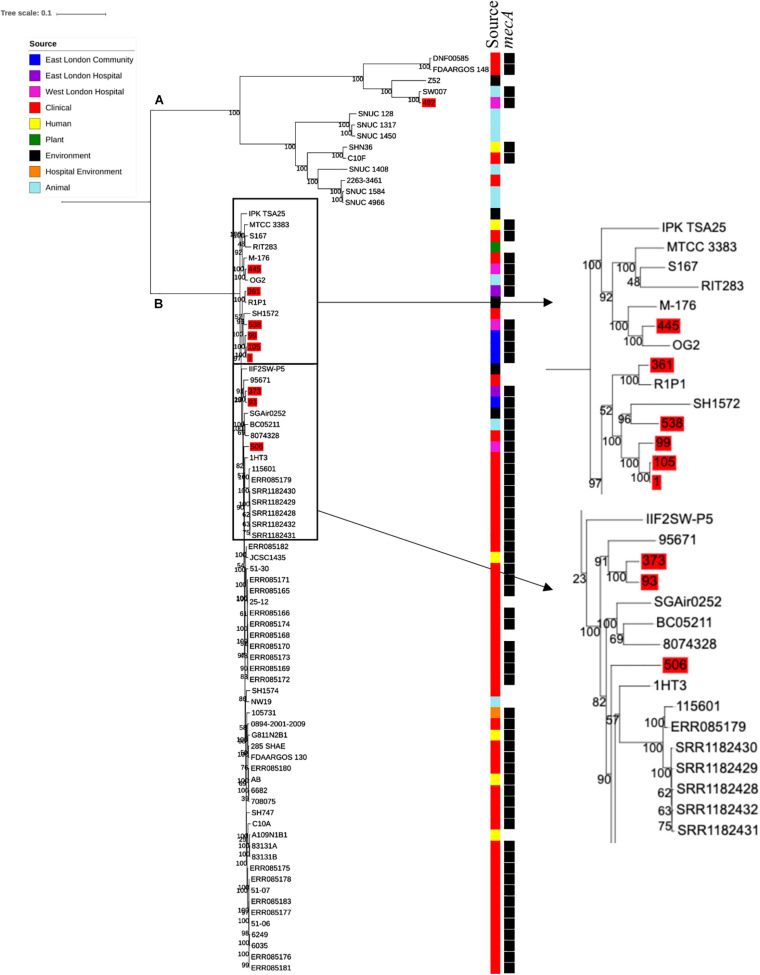
Core SNP maximum likelihood phylogenetic tree of 83 *S. haemolyticus*, including isolates recovered from public settings in East and West London and those obtained from the ENA. **(A,B)** represent the two most distinct clades. Isolates recovered from public settings in London are highlighted in red.

Ten *S. haemolyticus mecA*^+^ isolates recovered from public settings in East and West London were phylogenetically compared with isolates recovered from other sources, including those recovered from different infections (*n* = 48) and central venous catheter (*n* = 2), commensal isolates recovered from human skin, nares and eyes (*n* = 7); livestock (cow; *n* = 7), kefir (fermented cow milk drink; *n* = 1), companion animals (dog; *n* = 1), public settings [from the surface area of a building and tropical air sample (*n* = 2)], natural environments (*n* = 3) and plant-associated isolates (*n* = 2). The core SNP maximum likelihood phylogenetic tree revealed two distinctive clades (clade A and clade B), of which 65 out of 83 isolates were identified as *mecA*^+^ (Figure 3). Clade A consisted of ENA isolates included those recovered from the bloodstream, vagina and sputum, livestock (cows), a companion animal (dog), groundwater and healthy human eyes. Clade B consisted of ENA isolates recovered from the bloodstream, eye infection, colon infection, central venous catheter, healthy human skin, kefir, willow tree, livestock (cows), tropical air samples, copper alloy coin, surface area of a building and waste and the hygiene compartment of the International Space Station. In addition, all *mecA*^+^ *S. haemolyticus* isolates recovered from public areas in London in our study were found in clade B, except for the isolate 492, which was recovered from (WLH). We found that isolates we recovered from public areas in ELH and WLH (373, 445 and 538) and isolates recovered from the public settings in East London (1, 93, 99 and 105) were genetically related to ENA isolates that have previously been recovered from an eye infection (SH1572), bloodstream (M-176) and central venous catheter (95671). In addition, one isolate recovered from public areas in WLH (492) in this study were genetically related to an ENA isolate that has previously been recovered from a dog (SW007), whereas one isolate (445) was genetically similar to an isolate that was recovered from kefir (OG2; obtained from the ENA). Moreover, we found four isolates obtained from the ENA, two livestock-associated isolates from cows (BC05211 and NW19), one isolate recovered from kefir (OG2) and one isolate recovered from a willow plant (RIT283) were genetically related to ENA isolates which were recovered from the bloodstream. Interestingly, using the ENA, we were unable to find *S. haemolyticus* isolates that belonged to the same genetic lineage of isolate 506, which was recovered in public areas in the hospital in West London in this study.

**FIGURE 4:**
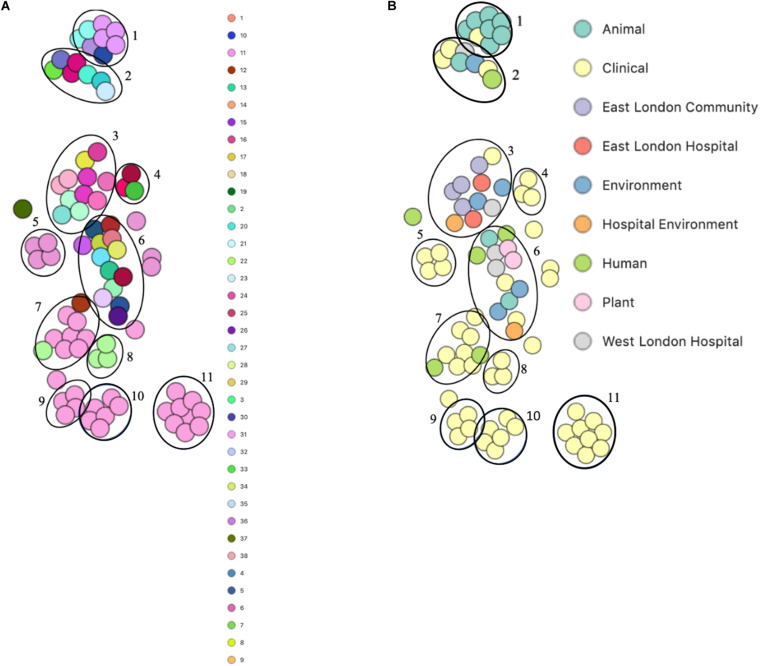
PoPpunk t-SNE analyses of accessory genomes of 83 *S. haemolyticus* isolates. Analyses were performed using PopPUNK pipeline. The maximum number of mixture components was set at 4 and for the perplexity of t-SNE set at 15. **(A)** Combined core and accessory genome cluster from PopPUNK analyses; **(B)** isolation source.

PopPUNK analyses revealed that there were 38 combined core and accessory gene clusters predicted, of which the accessory genome was found within 11 clusters (Figure 4). Five of these clusters included isolates that were identified belonging to the same combined cluster and six clusters included isolates that were only composed of those ENA isolates that have previously been recovered from clinical samples (blood). Interestingly, isolates recovered from the East London Community and the ELH were found together in the same cluster despite not always possessing the same combined core and accessory cluster (cluster 3). Isolates recovered from West London were found in different clusters (clusters 2, 3 and 6). We observed genetic relatedness of the accessory genomes of all isolates recovered from East London with those ENA isolates that have previously been recovered from an eye infection (SH1572); venous catheter (95671); and environmental isolates (a copper alloy coin; R1P1), whereas genetic relatedness of the accessory genomes of isolates recovered from West London was observed with those ENA isolates that were recovered from eye infections (SH1572 and SH1574), colon (1HT3), bloodstream (FDAARGOS-148), vagina (DNF00585) and sputum (C10F); healthy humans (JCSC1435, MTCC 3383 and SHN3) and plants (RIT283 and 167); livestock (NW19) and a companion animal (SW007).

### Phylogenetic Analysis of *S. hominis*

**FIGURE 5:**
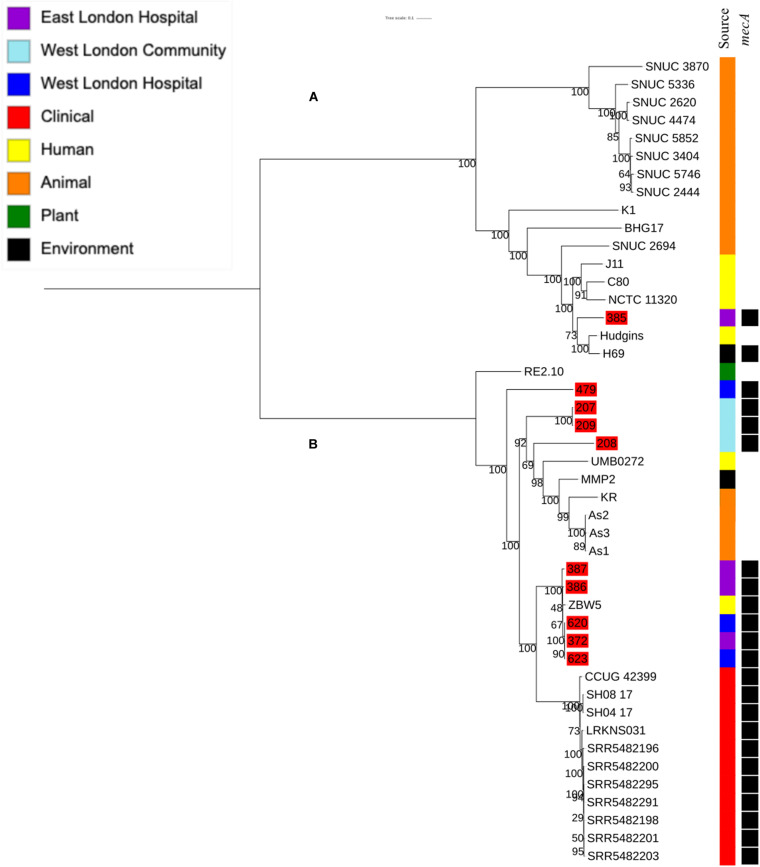
Core SNP maximum likelihood phylogenetic tree of 45 *S. hominis*, including isolates recovered from public settings in East and West London and those obtained from the ENA. **(A,B)** represent the two most distinct clades. Isolates recovered from public settings in London are highlighted in red.

To determine the relatedness of *S. hominis* isolates in this study with those in the ENA, 10 *mecA^+^ S. hominis* isolates recovered from public settings in East and West London in our study were phylogenetically compared with *S. hominis* isolates recovered from different sources that have previously been submitted to the ENA. This included isolates recovered from bloodstreams (*n* = 11), healthy human skin (*n* = 6); livestock (cows; *n* = 11), kefir (*n* = 1), mosquitoes (*n* = 3), natural environments (ancient permafrost and an air sample from residential areas; *n* = 2) and a plant isolate (rice seeds; *n* = 1). SNP core phylogenetic tree of *S. hominis* isolates contained two distinct clades: A and B (Figure 5). Clade A consisted of isolates obtained from the ENA and previously recovered from livestock (cows), healthy human skin, the air in residential areas, whereas clade B consisted of ENA isolates previously recovered from clinical bloodstream infections, healthy human skin, kefir and rice seeds, mosquitoes and ancient permafrost. Among *mecA^+^ S. hominis* isolates recovered in this study, only the isolate 385 from public areas in hospitals was found in clade A, whereas the remaining of the isolates were found together in clade B. Isolates 387, 386, 620, 623 and 372 recovered from hospitals in East and West London were found in the same subclade and were genetically related to ENA isolate that have been previously recovered from healthy human skin (ZBW5). Isolates 207, 208 and 209 recovered from West London public areas in the community have been found in the same subclade and were genetically related to an isolate recovered from a skin of a healthy human (UMB022), environmental isolates (ancient permafrost in Russia; MMP2), Asian Malaria Mosquito bodies (AS1, AS2 and AS3) and kefir (KR) (Hughes et al., 2016; Kashuba et al., 2017). We did, however, observe that isolates 207, 208 and 209 in this subclade had divergence in their genetic relationship with the ENA isolates. Isolate 385 recovered from East London was genetically related to ENA isolates previously recovered from healthy humans (Hudgins) and air samples from residential areas (H69). Interestingly, isolate 479 recovered from public areas in a WLH was not genetically related to other isolates. All *S. hominis* ENA isolates that were genetically related to isolates from public settings in London except the ENA isolate recovered from mosquito and the permafrost have been shown to harbour the *mecA* gene. All *S. hominis* ENA isolates that were previously recovered from clinical samples and 8 out of 10 ENA isolates recovered from livestock (SNUC 2444, SNUC 5746, SNUC 3403, SNUC 5852, SNUC 4474, SNUC 2620, SNUC 5336 and SNUC 3870) were not genetically related to isolates that we recovered from public settings or other ENA isolates we analysed.

**FIGURE 6:**
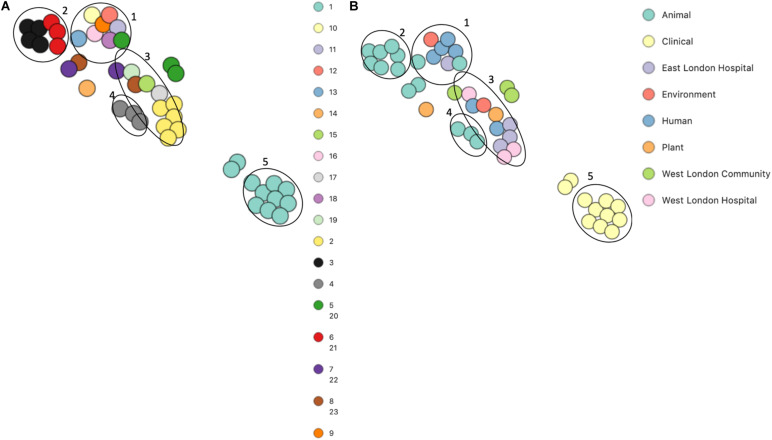
PoPpunk t-SNE analyses of the distance of the accessory genome in 45 *S. hominis* isolates. Analyses were performed using PopPUNK pipeline. The maximum number of mixture components was set at 5 and for the perplexity of t-SNE set at 15. **(A)** Combined core and accessory genome cluster from PopPUNK analyses: **(B)** isolation source.

PopPUNK analyses identified 23 combined core and accessory gene clusters, five of which were distinct clusters based on accessory genomes (Figure 6). Clinical isolates accessory genomes were found to be clustered together (cluster 5) distinct from those that were recovered from different sources. Additionally, 8 of the 11 *S. hominis* ENA isolates that have been previously recovered from livestock (SNUC 2444, SNUC 5746, SNUC 3403, SNUC 5852, SNUC 4474, SNUC 2620, SNUC 5336 and SNUC 3870) were clustered together (cluster 2). All but one (385) *mecA*^+^ isolates recovered from public settings in hospitals in this study, were in the same accessory genome cluster (cluster 4). This cluster includes isolate 479 from WLH, which was shown not to be phylogenetically related to other isolates by its core genome. The isolates in cluster 4 in this study were recovered from public settings and were related to those ENA isolates that have previously been recovered from healthy human skin (ZBW5), rice seed (RE2.10) and air samples from residential areas (H69) by their accessory genome.

### Accessory Gene Analysis of ENA of the *S. hominis* Clinical Isolates

Core genome phylogenetic analyses showed that *S. hominis* ENA isolates that were previously recovered from the hospital associated infections belonged to a single subclade and formed a single cluster by accessory genome analyses (PopPunk pipeline). Due to these observations, we decided to investigate these isolates further to identify whether they harboured any unique genes ubiquities to them/their accessory genome. We found 22 unique genes that were ubiquities to these *S. hominis* hospital associated infections ENA isolates. Fifteen of these genes were identified as being hypothetical (Supplementary Table 3). Those that were identified as non-hypothetical genes included the chromosome recombinase gene *ccrA3/B3*; transposition regulatory protein allele *tnpB*; cadmium resistance genes *cadA*, *cadX and cadD* allele; putative DNA repair protein, *radC* and copper-sensing transcriptional repressor *ricR*.

## Discussion

Genomic comparative studies are vital to aid our understanding of the relatedness of pathogenic bacteria recovered from different ecological niches and the transmission of these bacteria between human, livestock and the environment (Hartfield et al., 2014). To date, the majority of phylogenetic studies of CoNS has focused on studying bacteria recovered from clinical settings (Cavanagh et al., 2014; Post et al., 2017). In addition, there is a limited number of studies reporting on community-associated and livestock-associated CoNS, but little is known about the genetic lineages of CoNS recovered from public settings (Conlan et al., 2012; Chaudhry and Patil, 2016). Using a comparative genome approach, we aimed to determine whether the *mecA*^+^ CoNS recovered from public settings in East and West London in our study were genetically related to those isolates previously submitted to the ENA by others, including isolates recovered from different sources and those associated with infections (McEwen and Collignon, 2018).

In our study, we found that *S. epidermidis*, *S. haemolyticus* and *S. hominis* were the most common species of CoNS recovered from public settings that were *mecA*^+^. This is consistent with reports that previously showed that these species were the most common isolates recovered from nosocomial infections and public settings (Xu et al., 2015; Asaad et al., 2016; Seng et al., 2017). Given that there is a limited number of WGS data available in the ENA which has the record of their isolation source^4^ for *S. epidermidis*, *S. haemolyticus* and *S. hominis* isolated from different sources, we carried out phylogenetic analyses of these isolates only.

Core phylogenetic analysis of all three species revealed that *mecA* isolates recovered from these setting were genetically diverse and span across different clades. For *S. epidermidis the* majority of the isolates recovered from public settings in WLH (711, 712, 713, 715 and 716) were phylogenetically similar to ENA clinical isolates (bloodstream) by their core genome as well as their sequence types (ST2). The discovery of ST2 in public areas hospital areas is not surprising as it is the most common sequence types found in hospital-acquired infections (Deplano et al., 2016). However, it does suggest a route in which well-known hospital-acquired strains can reach the community from a public area. We also found that *mecA*^+^ isolates 435, 475 and 631 recovered from the public areas in WLH were genetically related to clinical isolates recovered from bloodstream; isolates 436 recovered from West London was genetically related to isolate recovered from urine tract infection (FDAARGOS-83), whereas isolate 465 was genetically related to an isolate recovered from endotracheal tube biofilm of a mechanically ventilated patient (ET-024). Additionally, ENA isolates Y24, PR246B0 M01 and M025 previously recovered from livestock and their housing was phylogenetically related to an isolate in this study that was recovered from public areas in hospitals (407). These isolates belonged to the same sequence type (ST59), which has previously been associated with isolates recovered from both livestock and humans (Argudín et al., 2015b; Xu et al., 2018a). These findings indicate that isolates found in public areas in hospitals are genetically related to those that have previously been reported to be associated with infections in humans and livestock. Other studies have shown *mecA*^+^ *S. epidermidis* as a common cause in bovine mastitis as well as has been recovered from cows milk (Fessler et al., 2010; Fernandes Dos Santos et al., 2016). In addition, pigs have also been shown to be a reservoir of *mecA*^+^ *S. epidermidis* that had similar virulence and antibiotic resistance gene profiles as isolates recovered from humans, largely indicating the transmission between humans and pigs (Tulinski et al., 2012; Argudín et al., 2015a). These reports, combined with our data, suggest that *S. epidermidis* may represent zoonoses and that livestock-associated *mecA*^+^ *S. epidermidis* isolates belong to the same genetic lineages as the isolates that have been shown to cause infections in humans. Some known lineages that cause infections in humans may have originated in animals and have been transferred to humans and their associated environments either via direct contact of farmers with animals or via food.

For *S. haemolyticus* core genome phylogenetic analysis all but two isolates recovered from public settings in this study were genetically related to isolates recovered from an eye conjunctivitis (SH1572), bloodstream infection (M-176) and central venous catheter (95671) obtained from the ENA. These results show that the isolates recovered from public settings in this study may potentially pose a public health risk as they belong to the same genetic lineages that have been shown to cause eye conjunctivitis and bloodstream infections (Cavanagh et al., 2014; Panda and Singh, 2016). This includes the four *mecA*^+^ isolates that were recovered from general public settings in East London (1, 93, 99 and 105), suggesting that isolates that cause bloodstream infections are not only present in public areas in hospitals but can also be found in general public settings. Moreover, we also identified that isolates recovered from public areas in hospitals were genetically related to those isolates that have previously been recovered from a dog (SW007; ENA) and kefir, that as a fermented milk product commonly contains different species of CoNS (OG2; ENA), which have been shown to carry the *mecA* gene (Prado et al., 2015). Previous reports have demonstrated that companion animals are potential reservoirs for the *mecA* gene, which can be transmitted to humans via contact as well as via food products (Ruzauskas et al., 2014). In this study, we were not able to determine whether those *S. haemolyticus* isolates that were genetically related to an isolate recovered from a dog could pose a potential risk to public health as no previous studies have linked the genetic lineages of *S. haemolyticus* isolates recovered from companion animals to that of isolates that have caused infections in humans. To the best of our knowledge, there are no reports that *S. haemolyticus* recovered from livestock belongs to the same genetic lineages known to cause infections in humans. In addition, we found that an isolate in the ENA recovered from kefir (OG2), was also related to the isolate in the ENA that has been recovered from clinical bloodstream infection (M-176).

Core genome phylogenetic analysis of *S. hominis* showed that the ENA livestock (cows) and ENA clinical isolates were genetically different to each other with none being related to the isolates recovered from public settings in East and West London suggesting that that the isolates from livestock and clinical samples have evolved separately and not crossed over into other niches. We did, however, find that our isolates from public setting to be phylogenetically related to isolates recovered from healthy humans skin (Hudgins and ZBW5), air samples from residential areas (H69), mosquitoes’ bodies (As1, As2 and As3), ancient permafrost (MMP2) and kefir (KR) (Hughes et al., 2016; Rivera-Perez et al., 2016). This suggests that mosquitoes could be possible vectors for transmitting *S. hominis* while feeding on their host and that genetically these *mecA*^+^ *S. hominis* isolates recovered from general public settings have not evolved much since ancient times (Hughes et al., 2016). Mosquitoes are vectors for viruses, protozoa and parasites that can spread and cause disease in humans and animals but currently, it is unreported if they can transfer and initiate bacterial infections (Torres-Guerrero et al., 2017; Assaid et al., 2020; Multini et al., 2020). The ENA isolates recovered from mosquitoes (As1, As2 and As3) were genetically related to an ENA isolate recovered from healthy human skin (UMB0272) (Figure 6). Therefore, we can conclude that the ENA isolates recovered from mosquitoes belong to the same genetic lineages as those recovered from humans. In addition, the findings that the *mecA*^+^ *S. hominis* isolates (385) in our study belonged to the same genetic lineage as the isolate recovered from an air sample in a residential area (H69) suggests that *S. hominis* can be transmitted through the air from humans to high-frequency touched surfaces or vice versa (Lymperopoulou et al., 2017).

Accessory genome is important for bacterial adaption and survival in different environments with studies of *Vibrio vulnificus, Legionella pneumophila* and *Pseudomonas aeruginosa* showing that clinical and environmental isolates can be distinguished by their accessory genomes (Kung et al., 2010; Koton et al., 2015; Mercante et al., 2018, p. 1). However, studies looking at accessory genomes of CoNS species recovered from different ecological niches are lacking. In the t-SNE plots, we observed that all the clusters generated in *S. epidermidis* analyses had a mixture of isolates from different sources, whereas *S. haemolyticus* and *S. hominis* analysis had clusters that were generated purely on isolates obtained from the ENA, which have been recovered from clinical bloodstream infection samples. In addition, there were two clusters of *S. hominis* ENA isolates that were only recovered from livestock (cows). This suggests that *S. epidermidis* isolates recovered from infections have previously been recovered from other niches. This also coincides with a previously published study which showed that *S. epidermidis* infections are derived from a diverse genetic backgrounds that possess *k*-mers (infection-associated genetic elements) associated with pathogenicity traits (Méric et al., 2018). These genes have likely originated from bacterial species associated with a particular niche and been transmitted to a new niche via humans or food. We did, however, observed clusters in the accessory genome that only contain *S. haemolyticus* and *S. hominis* ENA clinical associated isolates. This suggests that these isolates might have originated in nosocomial environments but have not spread to other non-hospital niches. This is further supported by the fact that *S. hominis* isolates in this study shared 22 unique genes with those isolates that have previously been recovered from different infections. We found that of these 22 genes, in particular those responsible for cadmium and copper resistance were the most interesting. Previous studies have shown the importance of cadmium resistance in *Helicobacter pylori* and *Listeria monocytogenes* virulence, whereas copper resistance is important for *S. aureus* survival within macrophages (Stähler et al., 2006; Purves et al., 2018). Moreover, it has been shown that metal resistance genes (cadmium, arsenic and zinc) are extensively exchanged between clinical associated *S. epidermidis* and *S. aureus* isolates and may play a role in their survival within hospital settings (Méric et al., 2015). However, it is currently unknown whether these genes contribute to the virulence or survival of *S. hominis* or *S. haemolyticus* within clinical settings. We found that *S. haemolyticus* isolates recovered from public settings in East London occupied the same cluster, whereas all but one *S. hominis* isolate from public areas in hospitals were found within the same cluster. This indicates that these species in these areas possess a similar pool of genes that are horizontally transferred due to the similarities in the microbiome in that geographical area or environment and/or adaption required to survive in these niches (Segerman, 2012). In addition, we found that *S. epidermidis* and *S. haemolyticus* recovered from public settings in this study had similar accessory genomes with those isolates that have previously been recovered from different infections (*S. epidermidis:* bloodstream, urinary tract infections and cerebrospinal fluid; *S. haemolyticus:* bloodstream and eye infections) healthy humans, animals (*S. epidermidis* and *S. haemolyticus:* livestock; *S. haemolyticus:* companion animals), plants and wider environments. Both core and accessory genomes phylogenetic analyses suggested that *mecA*^+^ isolates in this study originated from different ecological niches. Therefore, we hypothesis that these isolates contain genes associated with bacterial species across many different genera from different environments. These genes can horizontally be transferred to other CoNS isolates in public settings.

This study implies that CoNS bacteria from high frequency touched surfaces may be a potential risk to public health. Although, they lack virulence determinants, many cases of CoNS infections have been reported inside and outside of hospital settings (Rogers et al., 2009). In addition, CoNS harbour many types of antibiotic resistance genes which can horizontally be transferred across to more virulent pathogens (Xu et al., 2018b; Lee et al., 2019). It also plausible that the CoNS strains we recovered from public settings may have originated from animals either from livestock or companion animals. Animals can be a reservoir of antibiotic-resistant CoNS isolates which can be transmitted to humans furthering the spread of antibiotic-resistance bacteria (Bhargava and Zhang, 2012; Argudín et al., 2015a). The results of this study can be used to review the hand hygiene practices adapted by the general public; the design of the build environments to reduce infections and the use of appropriate disinfectants to prevent further transmission of potentially pathogenic bacteria in these areas.

## Conclusion

*MecA* positive *S. epidermidis* and *S. haemolyticus* isolates recovered from public settings in the community and hospitals in this study may pose a potential health risk as we showed that they belong to the same genetic lineages of those isolates that have previously been recovered from different infections (data obtained from the ENA). However, in this study we did not find similar features for the *S. hominis mecA*^+^ isolates recovered from public settings. In addition, we showed the relatedness of the *mecA*^+^ staphylococcal isolates (all three species) in this study with isolates recovered from livestock and healthy humans (ENA). Further studies are warranted to aid our understanding of whether the isolates recovered in this study that were genetically related to those recovered from livestock (ENA) could potentially cause infections in humans. The results from this study should be used to instigate a review on how the public wash their hands, the design of public areas (including ventilation practices) and how disinfectants are used to improve the hygiene practices in these areas.

## Data Availability Statement

The datasets presented in this study can be found in online repositories. The names of the repository/repositories and accession number(s) can be found in the article/ Supplementary Material.

## Author Contributions

RC: formal analysis, methodology, and writing – original draft. RM: data curation, formal analysis, methodology, software, and writing – review and editing. JC: methodology, resources, software, and writing – review and editing. SW: investigation and validation. HVM: conceptualization, data curation, funding acquisition, methodology, project administration, resources, supervision, and writing – review and editing. All authors contributed to the article and approved the submitted version.

## Conflict of Interest

The authors declare that the research was conducted in the absence of any commercial or financial relationships that could be construed as a potential conflict of interest.

## Footnotes

1 https://github.com/tseemann/barrnap

2 https://www.sanger.ac.uk/science/tools

3 https://github.com/edgardomortiz/vcf2phylip

4 https://www.ebi.ac.uk/ena
