# Supplementary material for: Comparative Genomics Analysis Demonstrated a Link Between Staphylococci Isolated From Different Sources: A Possible Public Health Risk
Source: Front Microbiol. 2021 Feb 25;12:576696. doi: 10.3389/fmicb.2021.576696 (PMC7947369; doi:10.3389/fmicb.2021.576696)
Supplement: Supplementary file 1 [file Data_Sheet_1.docx]

Supplementary Material

# Supplementary Figures and Tables

## Table S1: Summary of the whole genome sequenced isolates recovered from general public settings in East and West London

| **Accession No.** | **Isolate No** | **Species** | **Area isolated** |
| --- | --- | --- | --- |
| ERS2999996 (SAMEA5192505) | 1 | *Staphylococcus haemolyticus* | East London Community |
| ERS2999997 (SAMEA5192506) | 27 | *Staphylococcus sciuri* | East London Community |
| ERS2999998 (SAMEA5192507) | 33 | *Staphylococcus sciuri* | East London Community |
| ERS2999999 (SAMEA5192508) | 59 | *Staphylococcus sciuri* | East London Community |
| ERS3000000 (SAMEA5192509) | 74 | *Staphylococcus sciuri* | East London Community |
| ERS3000001 (SAMEA5192510) | 75 | *Staphylococcus sciuri* | East London Community |
| ERS3000002 (SAMEA5192511) | 93 | *Staphylococcus haemolyticus* | East London Community |
| ERS3000003 (SAMEA5192512) | 99 | *Staphylococcus haemolyticus* | East London Community |
| ERS3000004 (SAMEA5192513) | 105 | *Staphylococcus haemolyticus* | East London Community |
| ERS3000005 (SAMEA5192514) | 109 | *Staphylococcus sciuri* | East London Community |
| ERS3000006 (SAMEA5192515) | 207 | *Staphylococcus hominis* | West London Community |
| ERS3000007 (SAMEA5192516) | 208 | *Staphylococcus hominis* | West London Community |
| ERS3000008 (SAMEA5192517) | 209 | *Staphylococcus hominis* | West London Community |
| ERS3000009 (SAMEA5192518) | 211 | *Staphylococcus cohnii* | West London Community |
| ERS3000010 (SAMEA5192519) | 321 | *Staphylococcus epidermidis* | East London Hospital |
| ERS3000011 (SAMEA5192520) | 327 | *Staphylococcus epidermidis* | East London Hospital |
| ERS3000012 (SAMEA5192521) | 329 | *Staphylococcus epidermidis* | East London Hospital |
| ERS3000013 (SAMEA5192522) | 343 | *Staphylococcus cohnii* | East London Hospital |
| ERS3000014 (SAMEA5192523) | 349 | *Staphylococcus cohnii* | East London Hospital |
| ERS3000015 (SAMEA5192524) | 355 | *Staphylococcus epidermidis* | East London Hospital |
| ERS3000016 (SAMEA5192525) | 361 | *Staphylococcus haemolyticus* | East London Hospital |
| ERS3000017 (SAMEA5192526) | 372 | *Staphylococcus hominis* | East London Hospital |
| ERS3000018 (SAMEA5192527) | 373 | *Staphylococcus haemolyticus* | East London Hospital |
| ERS3000019 (SAMEA5192528) | 385 | *Staphylococcus hominis* | East London Hospital |
| ERS3000020 (SAMEA5192529) | 386 | *Staphylococcus hominis* | East London Hospital |
| ERS3000021 (SAMEA5192530) | 387 | *Staphylococcus hominis* | East London Hospital |
| ERS3000022 (SAMEA5192531) | 407 | *Staphylococcus epidermidis* | East London Hospital |
| ERS3000023 (SAMEA5192532) | 435 | *Staphylococcus epidermidis* | West London Hospital |
| ERS3000024 (SAMEA5192533) | 436 | *Staphylococcus haemolyticus* | West London Hospital |
| ERS3000025 (SAMEA5192534) | 445 | *Staphylococcus haemolyticus* | West London Hospital |
| ERS3000026 (SAMEA5192535) | 465 | *Staphylococcus epidermidis* | West London Hospital |
| ERS3000027 (SAMEA5192536) | 475 | *Staphylococcus epidermidis* | West London Hospital |
| ERS3000028 (SAMEA5192537) | 479 | *Staphylococcus hominis* | West London Hospital |
| ERS3000029 (SAMEA5192538) | 492 | *Staphylococcus haemolyticus* | West London Hospital |
| ERS3000030 (SAMEA5192539) | 506 | *Staphylococcus haemolyticus* | West London Hospital |
| ERS3000031 (SAMEA5192540) | 538 | *Staphylococcus haemolyticus* | West London Hospital |
| ERS3000032 (SAMEA5192541) | 620 | *Staphylococcus hominis* | West London Hospital |
| ERS3000033 (SAMEA5192542) | 623 | *Staphylococcus hominis* | West London Hospital |
| ERS3000034 (SAMEA5192543) | 631 | *Staphylococcus epidermidis* | West London Hospital |
| ERS3000035 (SAMEA5192544) | 664 | *Staphylococcus epidermidis* | West London Hospital |
| ERS3000036 (SAMEA5192545) | 673 | *Staphylococcus epidermidis* | West London Hospital |
| ERS3000037 (SAMEA5192546) | 699 | *Staphylococcus warneri* | West London Hospital |
| ERS3000038 (SAMEA5192547) | 700 | *Staphylococcus warneri* | West London Hospital |
| ERS3000039 (SAMEA5192548) | 702 | *Staphylococcus warneri* | West London Hospital |
| ERS3000040 (SAMEA5192549) | 711 | *Staphylococcus epidermidis* | West London Hospital |
| ERS3000041 (SAMEA5192550) | 712 | *Staphylococcus epidermidis* | West London Hospital |
| ERS3000042 (SAMEA5192551) | 713 | *Staphylococcus epidermidis* | West London Hospital |
| ERS3000043 (SAMEA5192552) | 715 | *Staphylococcus epidermidis* | West London Hospital |
| ERS3000044 (SAMEA5192553) | 716 | *Staphylococcus epidermidis* | West London Hospital |

**Table S2: Summary of the isolates used for phylogenetic analyses**

| **Isolate name** | **Species** | **Accession number** | **Source** |
| --- | --- | --- | --- |
| SE2.9 | *S. epidermidis* | JRVN01000000 | Rice seed |
| SE4.7 | *S. epidermidis* | JRVP01000000 | Rice seed |
| SE4.6 | *S. epidermidis* | JRVO01000000 | Rice seed |
| SE4.8 | *S. epidermidis* | JRVQ01000000 | Rice seed |
| UC7032 | *S. epidermidis* | ARWU01000000 | Cured meat |
| CIM40 | *S. epidermidis* | ATCW02000000 | Mouse skin |
| APO27 | *S. epidermidis* | ATCU02000000 | Mouse skin |
| CIM28 | *S. epidermidis* | ATDF02000000 | Mouse skin |
| APO35 | *S. epidermidis* | ATCV02000000 | Mouse |
| NIHLM057 | *S. epidermidis* | AKGO01000000 | Human Occiput |
| VCU128 | *S. epidermidis* | AHLI01000000 | Clinical airways |
| NW32 | *S. epidermidis* | LJIF01000000 | Cow milk |
| NIHLM015 | *S. epidermidis* | AKGZ01000000 | Human |
| NIHLM037 | *S. epidermidis* | AKGT01000000 | Human |
| 14.1.R1 | *S. epidermidis* | [CP018842](https://www.ncbi.nlm.nih.gov/nuccore/CP018842.1) | Human skin |
| MRSE 52-2 | *S. epidermidis* | NTLC01000000 | Human nasopharynx |
| NIHLM023 | *S. epidermidis* | AKGU01000000 | Human toe web |
| S2 005 003 R3 50 | *S. epidermidis* | QFPG01000000 | Hospital surfaces and sink |
| M01 | *S. epidermidis* | LYWE01000000 | Cowhouse |
| ZSC | *S. epidermidis* | PHHR01000000 | Groundwater |
| y24 | *S. epidermidis* | NRSY01000000 | Bovine mastitis milk |
| PR246B0 | *S. epidermidis* | PCFD01000000 | Pig rectum |
| M25 | *S. epidermidis* | LYWF01000000 | Cowhouse |
| FDAARGOS-161 | *S. epidermidis* | CP014132 | Clinical Peripheral blood |
| 1457 | *S. epidermidis* | CP020463 | Central venous catheter |
| AG42 | *S. epidermidis* | JNLI01000000 | Animal Sheep rumen |
| Scl25 | *S. epidermidis* | ATDC02000000 | Mouse skin |
| CSF41498 | *S. epidermidis* | CP030246 | Clinical cerebrospinal fluid (meningitis) |
| SNUT | *S. epidermidis* | LQRB01000000 | Toluene treated bioreactor sludge |
| ATCC 12228 | *S. epidermidis* | CP022247 | Human skin and mucosal |
| PM221 | *S. epidermidis* | HG813242 | Cow |
| SNUC 5038 | *S. epidermidis* | PYYR01000000 | Cow |
| SNUC 75 | *S. epidermidis* | PYZF01000000 | Cow |
| FDAARGOS-83 | *S. epidermidis* | JTAY02000000 | Clinical (urine) |
| RP62A | *S. epidermidis* | CP000029 | Clinical (intravascular catheter-associated sepsis) |
| SRR1182420 | *S. epidermidis* | SRR1182420 | Clinical blood |
| ET-024 | *S. epidermidis* | JGVL01000000 | Endotracheal tube biofilm of a mechanically ventilated patient |
| SRR1182422 | *S. epidermidis* | SRR1182422 | Clinical (blood) |
| SRR1182424 | *S. epidermidis* | SRR1182424 | Clinical (blood) |
| SRR1182423 | *S. epidermidis* | SRR1182423 | Clinical (blood) |
| SRR1182419 | *S. epidermidis* | SRR1182419 | Clinical (blood) |
| SRR1182413 | *S. epidermidis* | SRR1182413 | Clinical (blood) |
| VCU037 | *S. epidermidis* | AFTY01000000 | Clinical human airways |
| M0881 | *S. epidermidis* | AOAJ01000000 | Human Skin |
| VCU045 | *S. epidermidis* | AFEI01000000 | Clinical (human airways) |
| FDAARGOS 153 | *S. epidermidis* | CP014119 | Clinical (peripheral blood) |
| 764 SEPI | *S. epidermidis* | JUTX01000000 | Clinical |
| M0026 | *S. epidermidis* | JBVX01000000 | Clinical (blood) |
| NIH06004 | *S. epidermidis* | AKHH01000000 | Clinical(blood) |
| NIH08001 | *S. epidermidis* | AKHG01000000 | Clinical (blood) |
| SRR1182399 | *S. epidermidis* | SRR1182399 | Clinical (blood) |
| SRR1182398 | *S. epidermidis* | SRR1182398 | Clinical (blood) |
| SRR1182400 | *S. epidermidis* | SRR1182400 | Clinical (blood) |
| SRR1182371 | *S. epidermidis* | SRR1182371 | Clinical (blood) |
| SRR1182410 | *S. epidermidis* | SRR1182410 | Clinical (blood) |
| SRR1182401 | *S. epidermidis* | SRR1182401 | Clinical (blood) |
| SRR1182412 | *S. epidermidis* | SRR1182412 | Clinical (blood) |
| SRR1182418 | *S. epidermidis* | SRR1182418 | Clinical (blood) |
| SRR1182416 | *S. epidermidis* | SRR1182416 | Clinical (blood) |
| BPH0662 | *S. epidermidis* | LT571449 | Clinical |
| DAR1907 | *S. epidermidis* | CP013943 | Clinical (blood) |
| ENVH131 | *S. epidermidis* | LYVR01000000 | Hospital environment |
| ENVH150 | *S. epidermidis* | LYVW01000000 | Hospital environment |
| LRKNS114 | *S. epidermidis* | LZEO01000000 | Hospital environment |
| LRKNS116 | *S. epidermidis* | LZEQ01000000 | Hospital environment |
| LRKNS117 | *S. epidermidis* | LZER01000000 | Hospital environment |
| SH06 17 | *S. epidermidis* | PHKN01000000 | Clinical (blood) |
| SH03_17 | *S. epidermidis* | PHKH01000000 | Clinical (blood) |
| SH07 17 | *S. epidermidis* | PHKM01000000 | Clinical (blood) |
| 760 SEPI | *S. epidermidis* | JUUB01000000 | Clinical |
| SNUC 3608 | *S. epidermidis* | QXSP01000000 | Cow |
| SNUC 901.1 | *S. epidermidis* | PYYQ01000000 | Cow |
| B45679-10 | *S. epidermidis* | MVFV01000000 | Clinical (blood) |
| FDAARGOS 148 | *S. haemolyticus* | LORN02000000 | Clinical (blood) |
| DNF00585 | *S. haemolyticus* | JRNK01000000 | Clinical (vagina) |
| Z52 | *S. haemolyticus* | PHHQ01000000 | Groundwater |
| SW007 | *S. haemolyticus* | MTIZ01000000 | Dog |
| SNUC 128 | *S. haemolyticus* | PZIV01000000 | Cow |
| SNUC 1317 | *S. haemolyticus* | PZIP01000000 | Cow |
| SNUC 1450 | *S. haemolyticus* | PZIL01000000 | Cow |
| C10F | *S. haemolyticus* | JQHA01000000 | Clinical (sputum) |
| SNUC 1408 | *S. haemolyticus* | PZIM01000000 | Cow |
| 2263-3461 | *S. haemolyticus* | CUEO01000000 | Clinical (teat) |
| SNUC 4966 | *S. haemolyticus* | PZIC01000000 | Cow |
| SNUC 1584 | *S. haemolyticus* | PZIK01000000 | Cow |
| IPK TSA25 | *S. haemolyticus* | NDWY01000000 | Surface area of a building with less than 200 occupants |
| M-176 | *S. haemolyticus* | CUEQ01000000 | Clinical blood |
| OG2 | *S. haemolyticus* | NCXH01000000 | Kefir |
| RIT283 | *S. haemolyticus* | JFOJ01000000 | Willow |
| S167 | *S. haemolyticus* | CP013911 | Leaf vegetable |
| MTCC 3383 | *S. haemolyticus* | LILF01000000 | Human |
| IIF2SW-P5 | *S. haemolyticus* | MIZW01000000 | waste and hygiene compartment of International Space Station |
| R1P1 | *S. haemolyticus* | AJVA01000000 | Copper alloy coin |
| SH1752 | *S. haemolyticus* | LRHN01000000 | Clinical infected eye |
| 95671 | *S. haemolyticus* | CUFA01000000 | Central venous catheter |
| SGAir0252 | *S. haemolyticus* | CP025031 | Tropical air samples collected in Singapore |
| SHN36 | *S. haemolyticus* | LRBN01000000 | Healthy eye |
| BC05211 | *S. haemolyticus* | MRUZ01000000 | Bovine milk |
| 8074328 | *S. haemolyticus* | CUFG01000000 | Clinical blood |
| 1HT3 | *S. haemolyticus* | LAKG01000000 | Clinical Colon |
| 115601 | *S. haemolyticus* | CUHH01000000 | Central venous catheter |
| ERR085179 | *S. haemolyticus* | ERR085179 | Clinical |
| SRR1182430 | *S. haemolyticus* | SRR1182430 | Clinical |
| SRR1182429 | *S. haemolyticus* | SRR1182429 | Clinical |
| SRR1182428 | *S. haemolyticus* | SRR1182428 | Clinical |
| SRR1182432 | *S. haemolyticus* | SRR1182432 | Clinical |
| SRR1182431 | *S. haemolyticus* | SRR1182431 | Clinical |
| ERR085182 | *S. haemolyticus* | ERR085182 | Clinical |
| JCSC1435 | *S. haemolyticus* | NC_007168 | Human |
| 51-30 | *S. haemolyticus* | CUDO01000000 | Clinical (blood) |
| ERR085171 | *S. haemolyticus* | ERR085171 | Clinical |
| 25-12 | *S. haemolyticus* | CUCI01000000 | Clinical (blood) |
| ERR085165 | *S. haemolyticus* | ERR085165 | Clinical |
| ERR085166 | *S. haemolyticus* | ERR085166 | Clinical |
| ERR085174 | *S. haemolyticus* | ERR085174 | Clinical |
| ERR085170 | *S. haemolyticus* | ERR085170 | Clinical |
| ERR085168 | *S. haemolyticus* | E RR085168 | Clinical |
| ERR085173 | *S. haemolyticus* | ERR085173 | Clinical |
| ERR085169 | *S. haemolyticus* | ERR085169 | Clinical |
| ERR085172 | *S. haemolyticus* | ERR085172 | Clinical |
| SH1574 | *S. haemolyticus* | LRBM01000000 | Clinical eye |
| NW19 | *S. haemolyticus* | MRUY01000000 | Bovine milk |
| SH747 | *S. haemolyticus* | LRHM01000000 | Clinical eye |
| 105731 | *S. haemolyticus* | CUHI01000000 | Catheter |
| 0894-2001-2009 | *S. haemolyticus* | QVPX01000000 | Umbilical wound |
| G811N2B1 | *S. haemolyticus* | PGWX01000000 | Human nares |
| 285 SHAE | *S. haemolyticus* | JVMX01000000 | Clinical |
| FDAARGOS_130 | *S. haemolyticus* | LOSE02000000 | Clinical |
| ERR085180 | *S. haemolyticus* | ERR085180 | Clinical |
| 708075 | *S. haemolyticus* | CUFF01000000 | Clinical |
| AB | *S. haemolyticus* | CUEN01000000 | Human nares |
| 6682 | *S. haemolyticus* | CUGF01000000 | Clinical blood |
| C10A | *S. haemolyticus* | JPRW01000000 | Clinical sputum |
| A109N1B1 | *S. haemolyticus* | PGWY01000000 | Human nares |
| 83131B | *S. haemolyticus* | CP025396 | Clinical |
| 83131A | *S. haemolyticus* | CP024809 | Clinical |
| ERR085175 | *S. haemolyticus* | ERR085175 | Clinical |
| ERR085178 | *S. haemolyticus* | ERR085178 | Clinical |
| 51-06 | *S. haemolyticus* | CUCU01000000 | Clinical (blood) |
| ERR085183 | *S. haemolyticus* | ERR085183 | Clinical |
| 51-07 | *S. haemolyticus* | CUCV01000000 | Clinical (blood) |
| ERR085177 | *S. haemolyticus* | ERR085177 | Clinical |
| ERR085176 | *S. haemolyticus* | ERR085176 | Clinical |
| ERR085181 | *S. haemolyticus* | ERR085181 | Clinical |
| 6035 | *S. haemolyticus* | CUFD01000000 | Clinical (blood) |
| 6249 | *S. haemolyticus* | CUFE01000000 | Clinical (blood) |
| SNUC 3870 | *S. hominis* | QXVR01000000 | Cow |
| SNUC 5336 | *S. hominis* | PZHX01000000 | Cow |
| SNUC 4474 | *S. hominis* | QXVP01000000 | Cow |
| SNUC 2620 | *S. hominis* | PZIA01000000 | Cow |
| SNUC 5852 | *S. hominis* | PZHV01000000 | Cow |
| SNUC 3404 | *S. hominis* | PZHY01000000 | Cow |
| SNUC 2444 | *S. hominis* | PZIB01000000 | Cow |
| SNUC 5746 | *S. hominis* | PZHW01000000 | Cow |
| K1 | *S. hominis* | MWPJ01000000 | Bovine milk |
| BHG17 | *S. hominis* | MPNR01000000 | Goose droppings |
| SNUC 2694 | *S. hominis* | PZHZ01000000 | Cow |
| H69 | *S. hominis* | LVVO01000000 | Air from residential area |
| Hudgins | *S. hominis* | MAYR01000000 | Human skin |
| J11 | *S. hominis* | FBVJ01000000 | Human |
| C80 | *S. hominis* | ACRM01000000 | Human |
| NCTC_11320 | *S. hominis* | PPQE01000000 | Human |
| RE2.10 | *S. hominis* | LWJR01000000 | Rice seed |
| UMB0272 | *S. hominis* | PKIP01000000 | Human |
| MMP2 | *S. hominis* | LNTW01000000 | Ancient permafrost |
| KR | *S. hominis* | NGVM01000000 | Kefir |
| As2 | *S. hominis* | LFKR01000000 | Whole mosquito body |
| As3 | *S. hominis* | LFKS01000000 | Whole mosquito body |
| As1 | *S. hominis* | LFKQ01000000 | Whole mosquito body |
| ZBW5 | *S. hominis* | AKGC01000000 | Human skin |
| CCUG 42399 | *S. hominis* | PPQX01000000 | Clinical (blood) |
| SH04_17 | *S. hominis* | PHKJ01000000 | Clinical (blood) |
| SH08_17 | *S. hominis* | PHKL01000000 | Clinical (blood) |
| LRKNS031 | *S. hominis* | LXRS01000000 | Clinical |
| SRR5482196 | *S. hominis* | SRR5482196 | Clinical (blood) |
| SRR5482200 | *S. hominis* | SRR5482200 | Clinical (blood) |
| SRR5482295 | *S. hominis* | SRR5482295 | Clinical (blood) |
| SRR5482291 | *S. hominis* | SRR5482291 | Clinical (blood) |
| SRR5482198 | *S. hominis* | SRR5482198 | Clinical (blood) |
| SRR5482201 | *S. hominis* | SRR5482201 | Clinical (blood) |
| SRR5482203 | *S. hominis* | SRR5482203 | Clinical (blood) |

**Table S3. 22 unique genes that were ubiquities to *S. hominis* isolates obtained from the ENA**

| Gene | Functions |
| --- | --- |
| *cadA* | cadmium-transporting ATPase, c*adA* |
| *ccrB3* | cassette chromosome recombinase B3 |
| group_3863 | hypothetical protein |
| group_3871 | hypothetical protein |
| group_3872 | hypothetical protein |
| group_3873 | hypothetical protein |
| group_3874 | hypothetical protein |
| group_3875 | hypothetical protein |
| *ccrA3* | cassette chromosome recombinase A3 |
| group_3878 | hypothetical protein |
| group_3879 | hypothetical protein |
| group_3880 | hypothetical protein |
| group_3881 | hypothetical protein |
| group_3882 | hypothetical protein |
| group_3883 | putative DNA repair protein, *radC* |
| *cadX* | putative cadmium efflux system accessory protein |
| *cadD* | cadmium resistance permease, cadD |
| *ricR* | Copper-sensing transcriptional repressor RicR |
| group_3890 | rhodanese-like domain-containing protein |
| group_3901 | hypothetical protein |
| group_3949 | hypothetical protein |
| t*npB* | transposition regulatory protein TnpB_1 |

**
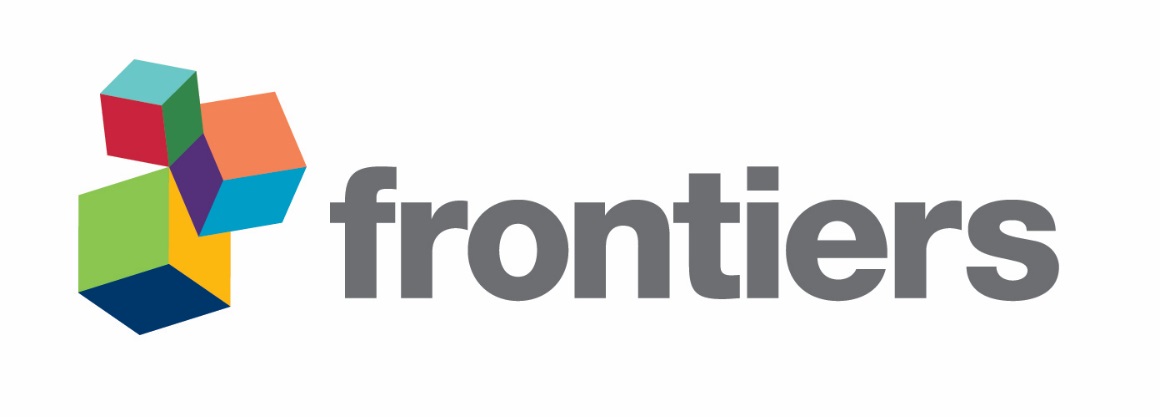
**
